# Theoretical and Experimental Study of Heterodyne Phase-Sensitive Dispersion Spectroscopy with an Injection-Current-Modulated Quantum Cascade Laser

**DOI:** 10.3390/s20216176

**Published:** 2020-10-29

**Authors:** Zhen Wang, Kin-Pang Cheong, Mingsheng Li, Qiang Wang, Wei Ren

**Affiliations:** 1 Department of Mechanical and Automation Engineering, The Chinese University of Hong Kong, New Territories, Hong Kong SAR, China; wangzhen@link.cuhk.edu.hk; 2 Shenzhen Research Institute, The Chinese University of Hong Kong, New Territories, Hong Kong SAR, China; 3 School of Aeronautics and Astronautics, Sichuan University, Chengdu 610017, China; kpcheong@scu.edu.cn; 4 Department of Biomedical Engineering, City University of Hong Kong, Kowloon, Hong Kong SAR, China; mingsheli2-c@my.cityu.edu.hk; 5 State Key Laboratory of Applied Optics, Changchun Institute of Optics, Fine Mechanics and Physics, Chinese Academy of Sciences, Changchun 130033, China; wangqiang@ciomp.ac.cn

**Keywords:** laser dispersion spectroscopy, quantum cascade laser, heterodyne detection, frequency modulation

## Abstract

We report the theoretical and experimental study of calibration-free heterodyne phase-sensitive dispersion spectroscopy (HPSDS) in the mid-infrared using a direct current modulated mid-infrared quantum cascade laser (QCL). The modulation of QCL current at several hundred MHz or higher generates the synchronous frequency and intensity modulation of the QCL emission. An analytical model of the phase of the beat note signal in HPSDS is derived by considering the absorption and dispersion processes and incorporating the QCL modulation parameters. In the experiment, a 4.5 μm QCL modulated at 350 MHz was used to measure N_2_O at 200 Torr in a 10 cm gas cell. The N_2_O concentrations inferred from the analytical model were compared with the nominal values to show good agreement over the concentration range of 189−805 ppm with a standard deviation <3%. When the QCL wavelength was locked at the line-center of the molecular transition, it was of interest to find that the theoretical model was simplified to that used for near-infrared HPSDS with an electro-optical modulator for laser modulation.

## 1. Introduction

Molecular dispersion spectroscopy has received more attention in the recent decade by measuring the refractive index in the vicinity of a molecular resonance [1,2,3,4]. Compared with absorption spectroscopy, it provides the advantages of inherent immunity to laser power fluctuations and large dynamic range [5]. Dispersion spectroscopy has been used in many applications such as remote open-path gas sensing [6,7,8], isotope analysis [9], and combustion diagnostics [10,11].

Representative detection schemes adopted for laser dispersion spectroscopy include chirped laser dispersion spectroscopy (CLaDS) [1], heterodyne phase-sensitive dispersion spectroscopy (HPSDS) [2], and multi-heterodyne dispersion spectroscopy [12,13]. Additionally, dispersion spectroscopy enhanced by using a high-finesse optical cavity, known as noise-immune cavity-enhanced optical-heterodyne molecular spectroscopy (NICE-OHMS), has achieved a sensitivity of 5 × 10^−13^ of integrated absorption [4]. Among these detection schemes, HPSDS is an attractive technique that features a simpler optical configuration and signal processing. In the previous studies of near-infrared HPSDS, a two- or three-color laser beam was generated by modulating laser intensity using commercial electro-optical modulators (EOMs). The refractive index of gas medium is then measured by phase-sensitive detection using a lock-in amplifier. The spectroscopic principle, theoretical model, and experimental setup for near-infrared HPSDS were well documented elsewhere [2,5]. Besides that, the MEMS-based vertical-cavity surface-emitting laser (VCSEL) was also applied in HPSDS for multispecies detection due to the mode-hop-free broadband wavelength tunability of the MEMS-VCSEL [3]. Compared with near-infrared gas sensing, a higher sensitivity could be achieved in the mid-infrared domain where most gas molecules have the fundamental absorption bands. However, commercial EOMs are not always available in the mid-infrared that limits the further application of HPSDS.

Martín-Mateos et al. reported a HPSDS-based gas sensor using a mid-infrared light source based on the difference frequency generation (DFG) [14]. The sidebands of the mid-infrared light could be generated by modulating the pump laser using EOMs. However, the DFG system is complex due to the use of a high power pump laser and the phase matching associated with the nonlinear crystal. Later on, the mid-infrared HPSDS was performed using a directly modulated quantum cascade laser (QCL) for the successful CO detection at 4.59 µm [15]. As opposed to the near-infrared intensity modulation with an EOM, the injection current of the QCL was directly modulated using a radio frequency (RF) signal generator at a modulation frequency of hundreds of MHz. Note that the current modulation of QCL at such a high frequency generates the synchronous intensity modulation (IM) and frequency modulation (FM) [16,17,18]. Additionally, HPSDS measurements are also affected by the sideband ratio associated with the laser modulation frequencies and intrinsic properties of QCLs. Thus, a reliable spectroscopic model is required for QCL-based HPSDS towards the realization of calibration-free measurements of gas concentrations. Hangauer et al. [18,19] conducted a relatively comprehensive investigation of the E-field emission of the directly current-modulated QCL for CLaDS. Instead of performing frequency demodulation of the beat note signal in CLaDS, the HPSDS technique measures the phase of the beat note signal after the frequency down-conversion.

In this work, we introduce a calibration-free model for HPSDS using QCLs in the mid-infrared. The model construction starts with the E-field emission reported for CLaDS [18,19] because both dispersion techniques use the high-frequency injection-current-modulated QCL. The new model only contains the specific modulation characteristics of the laser source, the spectroscopic parameters obtained from the HITRAN database [20], and the gas properties to be determined. As a proof-of-principle, we experimentally performed the HPSDS detection of N_2_O using a distributed-feedback (DFB) QCL near 4.5 µm. The theoretical model can be further simplified to the model used for near-infrared HPSDS with an EOM when the QCL wavelength is locked to the line-center of the molecular transition.

## 2. Model

In QCL-based dispersion spectroscopy, a three-color laser beam is generated by directly modulating (angular frequency Ω) the injection current of the laser. Here we adopted the identical symbols and expressions to represent the QCL emission as those used in the references [18,19]. The IM index *m* (defined as the amplitude of IM divided by the total laser intensity) and the FM index *β* (defined as the amplitude of FM divided by the modulation frequency) are both assumed to be small enough (*m* « 1, *β* « 1). Thus, the QCL emission has the carrier (*E*_0_) centered at the optical angular frequency *ω* and two modulation sidebands (*E*_1_ and *E_−_*_1_) at *ω* ± Ω. The E-fields can be expressed by the following time-domain forms:

(1) $$E_{0}=\sqrt{P_{0}}\cos\left(\omega t\right),$$

(2) $$E_{1}=\frac{m}{4}\sqrt{P_{0}}\left\{\cos\left[\left(\omega +\Omega \right)t\right]+\frac{2\beta }{m}\cos\left[\left(\omega +\Omega \right)t-\theta \right]\right\},$$

(3) $$E_{-1}=\frac{m}{4}\sqrt{P_{0}}\left\{\cos\left[\left(\omega -\Omega \right)t\right]-\frac{2\beta }{m}\cos\left[\left(\omega -\Omega \right)t+\theta \right]\right\},$$

where *P*_0_ is the laser intensity, and *θ* is the FM–IM phase shift.

The laser beam is then transmitted through the gas sample where each wavelength component experiences the corresponding phase shift and attenuation because of the molecular dispersion and absorption. After passing through the gas medium with a path length of *L*, the transmitted beam can be expressed as

(4) $$E_{0}^{\prime }=A_{0}\cos\left(\omega t-\psi_{0}\right),$$

(5) $$E_{1}^{\prime }=A_{1}\left\{\cos\left[\left(\omega +\Omega \right)t-\psi_{1}\right]+\frac{2\beta }{m}\cos\left[\left(\omega +\Omega \right)t-\theta -\psi_{1}\right]\right\},$$

(6) $$E_{-1}^{\prime }=A_{-1}\left\{\cos\left[\left(\omega -\Omega \right)t-\psi_{-1}\right]-\frac{2\beta }{m}\cos\left[\left(\omega -\Omega \right)t+\theta -\psi_{-1}\right]\right\},$$

where

$$A_{k}=e^{-\frac{\alpha \left(\omega +k\Omega \right)L}{2}}{\left(\frac{m}{4}\right)}^{\left|k\right|}\sqrt{P_{0}},\;\psi_{k}=\frac{\left(\omega +k\Omega \right)L}{c}\left[n\left(\omega +k\Omega \right)-1\right],$$

and the coefficient *k* = −1, 0, 1; *n*(*ω*) and α(*ω*) are the refractive index and absorption coefficient, respectively; *c* is the speed of light in vacuum. The transmitted laser beam is detected by a square-law photodetector.

(7) $$I\propto {\left(E_{0}^{\prime }+E_{1}^{\prime }+E_{-1}^{\prime }\right)}^{2}.$$

The beat note signal between the carrier and two sidebands can be obtained by the first harmonic detection. The dispersion information is encoded in the phase of the beat note signal.

The refractive index and absorption coefficient obey the Kramers–Kronig relation [21].

(8) $$n\left(\omega \right)=1+\frac{c}{\pi }℘\int_{0}^{+\infty }\frac{\alpha \left(\omega^{\prime }\right)}{{\omega^{\prime }}^{2}-\omega^{2}}d\omega^{\prime },$$

where the symbol ℘ denotes the Cauchy principal value, which is determined by the pole of the integrand for numerical calculation. Here we assume a Voigt line-shape for calculating the absorption profile [22,23]. Finally, the measured phase of the beat note signal can be expressed as

(9) $$\begin{array}{l} \varphi =\tan^{-1}\{[2F\beta \sin\left(\psi_{0}-\psi_{1}-\theta \right)-2G\beta \sin\left(\psi_{-1}-\psi_{0}-\theta \right)+\\ \begin{array}{ll} & Fm\sin\left(\psi_{0}-\psi_{1}\right)+Gm\sin\left(\psi_{-1}-\psi_{0}\right)]/\\ & [2F\beta \cos\left(\psi_{0}-\psi_{1}-\theta \right)-2G\beta \cos\left(\psi_{-1}-\psi_{0}-\theta \right)+ \end{array}\\ \begin{array}{cc} & Fm\cos\left(\psi_{0}-\psi_{1}\right)+Gm\cos\left(\psi_{-1}-\psi_{0}\right)]\} \end{array} \end{array}$$

where the coefficient *F* = exp[−*α*(*ω* + Ω)*L* − *α*(*ω*)*L*] and *G* = exp[−*α*(*ω* − Ω)*L* − *α*(*ω*)*L*]. It should be noted that laser characterization is required to obtain *β*, *m*, and *θ*. Though the analytical expression is somewhat complex, it clearly shows the contribution from FM sidebands and IM sidebands, respectively.

**Figure 1:**
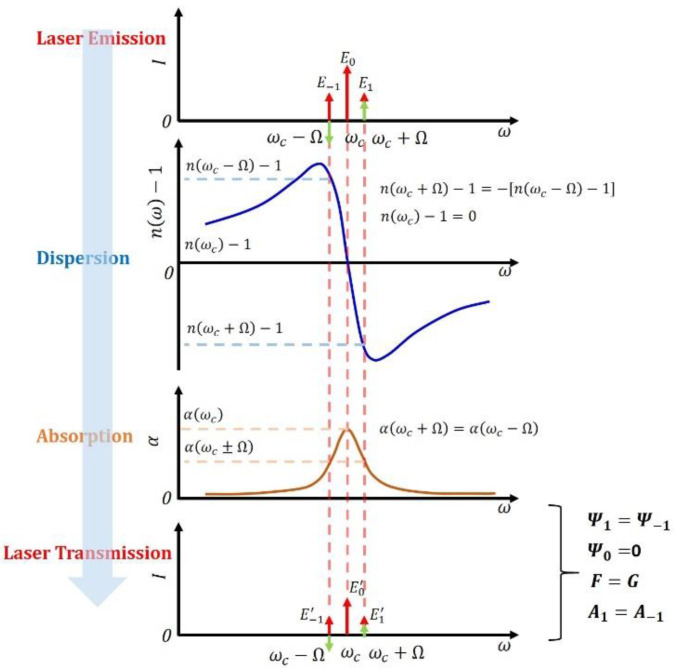
Schematic of the laser transmission through a gas sample due to the simultaneous absorption and dispersion. Red arrows: intensity modulation (IM) sidebands; green arrows: frequency modulation (FM) sidebands.

As the laser characterization process is not so straightforward, it is of interest to investigate the possible methods to obtain a simpler analytical expression. Here we assume the frequency (*ω*) of the laser carrier (*E*_0_) matches the line-center (*ω_c_*) of the gas molecule, as shown in Figure 1. Then the two pairs of sidebands induced by FM and IM have the equal frequency difference relative to the line-center of the molecular transition. Considering the symmetric line-shape of the absorption profile, both pairs of sidebands are attenuated equally so that *A*_1_ = *A*_−1_ in Equations (5) and (6), whereas the carrier experiences a larger attenuation. Thus the parameter *F* equals to parameter G in Equation (9). Considering the antisymmetric line-shape of the refractive index shown in Figure 1, the left sideband (*E*_1_′) and right sideband (*E_−_*_1_′) have the same modulus but an opposite phase shift, *Ψ*_1_ ≈ −*Ψ*_−1_. The following is assumed here:

(10) $$\psi_{k}=\frac{\left(\omega +k\Omega \right)L}{c}\left[n\left(\omega +k\Omega \right)-1\right]\approx \frac{\omega L}{c}\left[n\left(\omega +k\Omega \right)-1\right],$$

because *ω* ≫ Ω [2,5]. The carrier has no extra phase shift induced by the target molecule; thus, *Ψ*_0_ = 0.

Referring to the theoretical model, at the resonant frequency of the molecule, Equation (9) is simplified to the following expression:

(11) $$\varphi =\frac{\omega_{c}L}{c}\left[n\left(\omega_{c}+\Omega \right)-1\right].$$

Hence, the HPSDS phase signal at the resonant frequency of the gas molecule is only relevant to the gas properties, such as refractive index, but independent of laser parameters such as *β*, *m*, and *θ*.

We analyzed that such a simplification was mainly caused by the fact that FM sidebands are out of phase and IM sidebands are in phase, as shown in Equations (2) and (3). After experiencing the equal phase shift but with opposite signs, the beat notes between the carrier (Equation (4)) and the two FM sidebands (the latter parts of Equations (5) and (6)) cancel each other. The model becomes a pure intensity modulation that is similar to the near-infrared HPSDS using an EOM, because only the beat notes between carrier and the two IM sidebands remain in the model.

We can further verify this hypothesis by comparing the result with the near-infrared HPSDS. The measured phase of the beat note signal for the EOM-based near-infrared HPSDS is given by the following equation [2]:

(12) $$\varphi =\frac{\omega L}{2c}\left[n\left(\omega +\Omega \right)-n\left(\omega -\Omega \right)\right].$$

When *ω* = *ω_c_*, there exists the following relationship:

(13) $$n\left(\omega_{c}+\Omega \right)-1=-\left[n\left(\omega_{c}-\Omega \right)-1\right]$$

As a result, both HPSDS models converge to the same equation when the laser frequency is fixed at the line-center of the target absorption line. The HPSDS detection at the line-center provides a possible method to measure gas concentrations without the need for laser characterization.

## 3. Experimental

**Figure 2:**
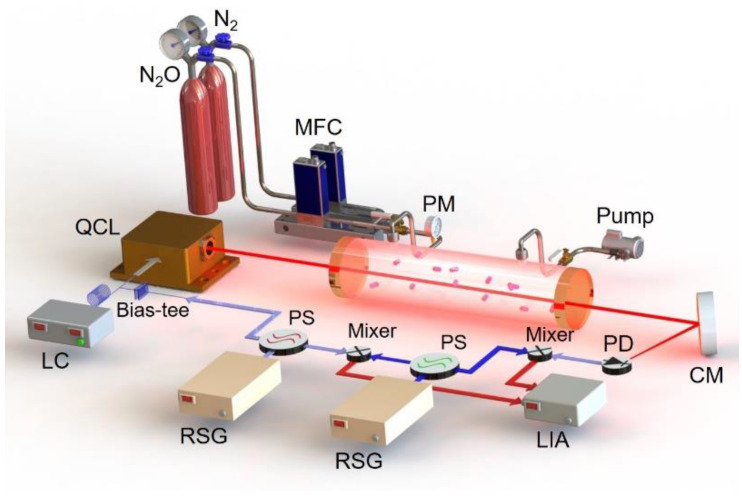
Schematic of the heterodyne phase-sensitive dispersion spectroscopy (HPSDS) gas sensing system. QCL, quantum cascade laser; LC, laser controller; RSG, RF signal generator; LIA, lock-in amplifier; PS, power splitter; PD, photodetector; CM, concave mirror; MFC, mass flow controller; PM, pressure meter.

To verify the theoretical model, a QCL-based HPSDS system was built with the configuration shown in Figure 2. A continuous-wave QCL (Alpes Lasers SA, St-Blaise in Switzerland) was used to exploit the R(18) line of N_2_O centered at 2238.36 cm^−1^. The QCL wavelength was scanned across the N_2_O absorption profile with a slow bias current ramp generated by a low-noise laser controller (Newport, Irvine, CA, USA, ILX LDC-3736). A high frequency (Ω/2π = 350 MHz) modulation signal from one channel of an RF generator (Stanford Research System, Sunnyvale, CA, USA, SG 382) was superimposed with the bias current via a bias-tee circuit, which was then injected to the QCL. Note that a power splitter was used to split the RF signal into two channels with the same frequency and initial phase. Hence, a three-color beam was generated by modulating the QCL injection current at a high modulation frequency.

The three-color beam was directed through a 10-cm gas cell filled with N_2_O/N_2_ mixtures at a fixed pressure of 200 Torr monitored by a pressure meter. The transmitted QCL beam impinged on a mercury cadmium telluride (MCT) photodetector (VIGO Systems, Ożarów Mazowiecki in Poland, PVI-4TE-10.6) with 800 MHz bandwidth to generate the heterodyne beat note signal. The detected electrical beat note signal (350 MHz) was then mixed with a sinusoidal waveform with a slightly different frequency (Ω/2π = 349.9 MHz) generated by another RF signal generator. Thus, the frequency of the beat note signal was downshifted to 100 kHz, which falls in the operation range of a commercial lock-in amplifier (Signal Recovery, Oak Ridge, TN, USA, Model 7265). Finally, the dispersion information was encoded in the phase of the frequency downshifted electrical signal that could be retrieved using the lock-in amplifier. To improve the measurement precision, the outputs of the two RF generators were mixed to generate the 100 kHz external reference for the lock-in amplifier.

## 4. Results and Discussion

**Figure 3:**
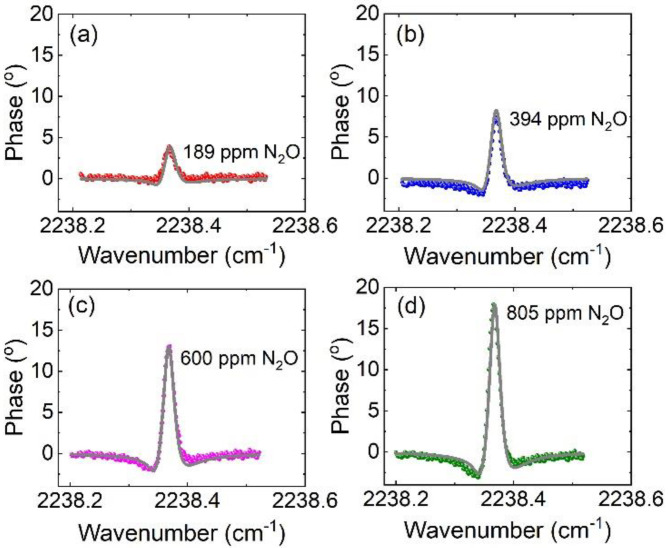
Representative HPSDS phase signals measured at 200 Torr for different N_2_O concentrations (symbol, experiment; line, simulation): (**a**) 189 ppm, (**b**) 394 ppm, (**c**) 600 ppm, and (**d**) 805 ppm.

Gas mixtures of N_2_O/N_2_ at different concentrations were prepared using a commercial gas dilution system (Jinwei Inc., Wuhan, China). Figure 3 presents the representative spectra of N_2_O measured by the HPSDS sensor at 200 Torr for four different concentrations. The slight asymmetry of the measured spectra was mainly due to the asymmetry of the sidebands generated by IM and FM of the QCL. The calculated HPSDS spectra based on the analytical model for the corresponding N_2_O concentrations are also plotted in Figure 3 for comparison. The spectral calculations were in good agreement with the measurements with a standard deviation <3%. Considering the standard deviation of the detection noise (1σ) of ~0.37°, we obtained a signal-to-noise ratio (SNR) of 12 for the N_2_O concentration of 189 ppm, corresponding to a minimum detectable N_2_O of 16 ppm using the current HPSDS sensor.

It should be noted that all the simulations were conducted using the characterized QCL parameters (*β*, *m*, and *θ*) and the known spectroscopic parameters of the R(18) line of N_2_O (line-strength and line broadening coefficients). The intrinsic QCL parameters *β*, *m*, and *θ* can be experimentally determined using direct measurement [18] or by fitting a HPSDS spectrum of known gas concentration with the analytical model. These parameters are found only associated with the bias current, modulation frequency, and modulation depth of the QCL.

**Figure 4:**
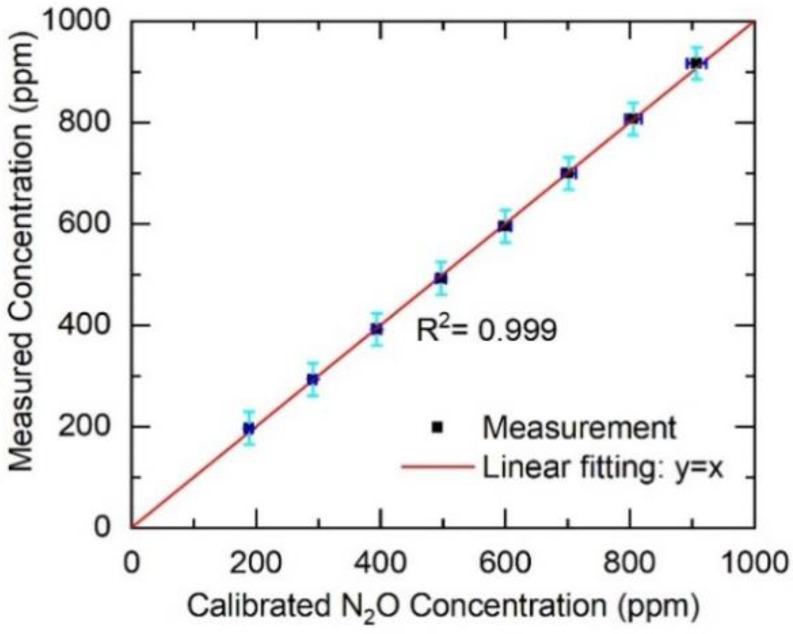
Comparison of the measured N_2_O concentration using HPSDS with the nominal concentration determined by the gas dilution system.

The peak-to-peak values of the measured dispersion spectra were compared with the model simulations to infer N_2_O concentrations. Figure 4 compares the HPSDS-determined N_2_O concentrations with the nominal concentrations determined by the gas dilution system. The horizontal error bars show the uncertainty of concentration values for dilutions by taking into account the uncertainty of flow meters. The uncertainties in line-strength and the detection noise of the sensor led to a measurement error bar (2σ) of 32 ppm in the N_2_O concentration. A linear relation (y = x) was obtained with an R-square value of 0.999, demonstrating the precision of the calibration-free HPSDS method.

**Figure 5:**
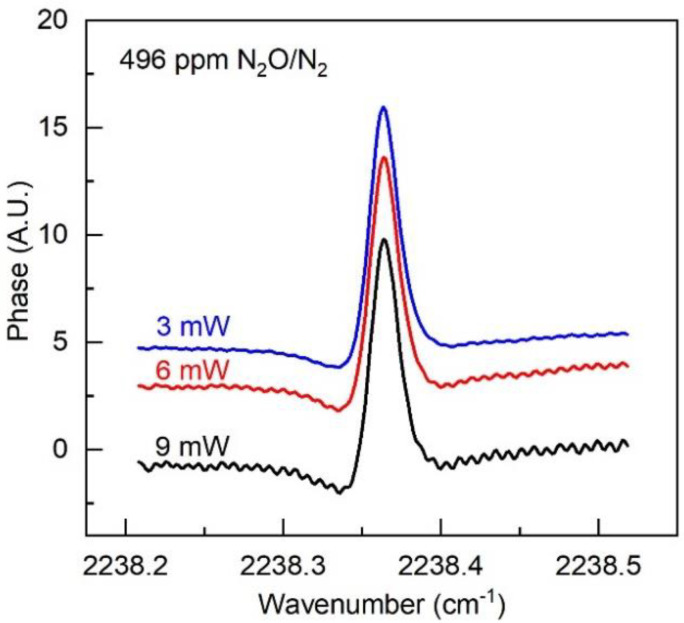
Measured HPSDS phase signals at different optical power levels for the same N_2_O concentration (496 ppm). The three signals are shifted vertically for viewing purposes.

As discussed previously, compared with absorption spectroscopy, dispersion spectroscopy has the advantage of intrinsic immunity to laser power fluctuations [2]. We thus investigated the HPSDS sensor performance by adjusting the laser power using an iris with all the other parameters unchanged. Figure 5 depicts the measured HPSDS phase signals for the same N_2_O mixture (496 ppm) at three different optical powers (3 mW, 6 mW, and 9 mW). These spectra show the negligible difference (<2.7%) of the peak-to-peak amplitude. In addition, we observed a slightly improved detection SNR at the lower laser power under the current experimental conditions.

**Figure 6:**
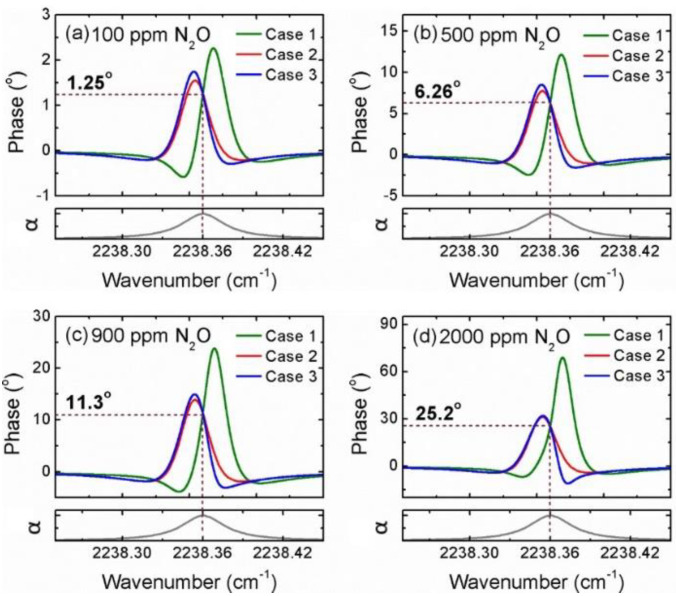
Calculated HPSDS spectra of N_2_O at (**a**) 100 ppm, (**b**) 500 ppm, (**c**) 900 ppm, and (**d**) 2000 ppm, for three different combinations of laser parameters *β*, *m*, and *θ*. Case 1: *β* = 0.004, *m* = 0.002, *θ* = 0.71π; case 2: *β* = 0.001, *m* = 0.002, *θ* = 0; and case 3: *β* = 0.0025, *m* = 0.002, *θ* = 0.31π.

Finally, based on the theoretical model in Equation (9), we performed detailed simulations to examine the influence of different laser parameters on HPSDS phase signals. In particular, we compared the three typical cases with different *β/m* ratios and *θ* values (case 1: *β* = 0.004, *m* = 0.002, *θ* = 0.71π; case 2: *β* = 0.001, *m* = 0.002, *θ* = 0; case 3: *β* = 0.0025, *m* = 0.002, *θ* = 0.31π). Note that the ratio of *β/m* affects the output phase more significantly than the individual parameter *β* or *m*. Figure 6 illustrates the simulation results of the HPSDS spectra for the four N_2_O concentrations (100 ppm, 500 ppm, 900 ppm, and 2000 ppm). The corresponding absorbance normalized by N_2_O concentration is plotted at the bottom panel of Figure 6. For each concentration, the three HPSDS signals with different combinations of *β*, *m*, and *θ* intersect at the same point, corresponding to the line-center of the absorption profile.

It should be noted that we could not experimentally verify Equation (11) using the current setup mainly due to the lack of appropriate locking techniques for the QCL to the molecular transition. Although the third-harmonic locking technique is commonly used in wavelength modulation spectroscopy [24], it modulates the laser frequency periodically at a low frequency that introduces unwanted phase fluctuations to the beat note signal. We believe that the high-frequency modulation like the Pound–Drever–Hall technique is a possible solution, as it may stabilize the carrier by narrowing the linewidth of QCL. However, it is out of the technical scope of the current study and will be further pursed in the future.

## 5. Conclusions

In conclusion, we performed the theoretical and experimental study of mid-infrared HPSDS using a directly current modulated QCL. To accurately simulate the detected HPSDS spectra, the IM and FM of QCL, the physical processes of absorption and dispersion, and the phase sensitive detection are considered in the analytical model. A final analytical model for the phase of the beat note signal in HPSDS is derived. We experimentally validated the theoretical model by detecting N_2_O at 200 Torr in a 10 cm long gas cell using a 4.5 μm DFB-QCL. The model simulations show good agreement with the experimental data. Although the laser characterization process is required for the current HPSDS model, we theoretically proved that a complete calibration-free measurement may be achieved by performing dispersion detection at the line-center of the molecular transition. Mid-infrared HPSDS provides an alternative gas sensing method with unique advantages compared with absorption spectroscopy.
